# Transforming Boolean models to continuous models: methodology and application to T-cell receptor signaling

**DOI:** 10.1186/1752-0509-3-98

**Published:** 2009-09-28

**Authors:** Dominik M Wittmann, Jan Krumsiek, Julio Saez-Rodriguez, Douglas A Lauffenburger, Steffen Klamt, Fabian J Theis

**Affiliations:** 1 Institute for Bioinformatics and Systems Biology, Helmholtz Zentrum München - German Research Center for Environmental Health, 85764 Neuherberg, Germany; 2 Department of Mathematical Science, Technische Universität München, 85747 Garching, Germany; 3 Biological Engineering Department, M.I.T., Cambridge MA 02139, USA; 4 Department of Systems Biology, Harvard Medical School, Boston MA 02115, USA; 5 Max Planck Institute for Dynamics of Complex Technical Systems, 39106 Magdeburg, Germany; 6 Max Planck Institute for Dynamics and Self-Organisation, 37073 Göttingen, Germany

## Abstract

**Background:**

The understanding of regulatory and signaling networks has long been a core objective in Systems Biology. Knowledge about these networks is mainly of qualitative nature, which allows the construction of Boolean models, where the state of a component is either 'off' or 'on'. While often able to capture the essential behavior of a network, these models can never reproduce detailed time courses of concentration levels.

Nowadays however, experiments yield more and more quantitative data. An obvious question therefore is how qualitative models can be used to explain and predict the outcome of these experiments.

**Results:**

In this contribution we present a canonical way of transforming Boolean into continuous models, where the use of multivariate polynomial interpolation allows transformation of logic operations into a system of ordinary differential equations (ODE). The method is standardized and can readily be applied to large networks. Other, more limited approaches to this task are briefly reviewed and compared. Moreover, we discuss and generalize existing theoretical results on the relation between Boolean and continuous models. As a test case a logical model is transformed into an extensive continuous ODE model describing the activation of T-cells. We discuss how parameters for this model can be determined such that quantitative experimental results are explained and predicted, including time-courses for multiple ligand concentrations and binding affinities of different ligands. This shows that from the continuous model we may obtain biological insights not evident from the discrete one.

**Conclusion:**

The presented approach will facilitate the interaction between modeling and experiments. Moreover, it provides a straightforward way to apply quantitative analysis methods to qualitatively described systems.

## Background

Close interaction between experiments and mathematical models has proven to be a powerful research approach in Systems Biology. Especially the modeling of regulatory and signaling networks, however, is typically hampered by a lack of information about mechanistic details, as often one can only determine the interactions of the involved species in a qualitative way. The current shift of focus in Systems Biology from single signal transduction pathways to networks of pathways exacerbates this lack of information even more. Therefore, the creation of mass action based models that accurately describe the underlying biochemistry is typically restricted to small well-studied subsystems.

**Figure 1:**
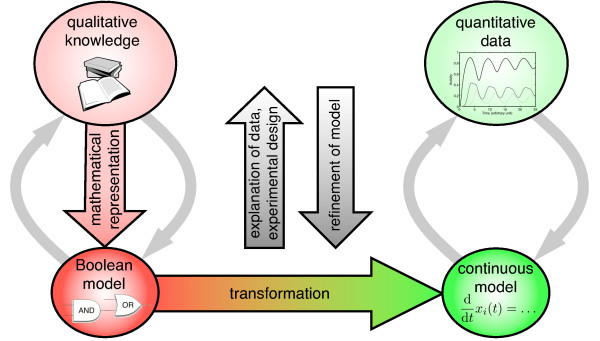
**Overview of the modeling process**. The typically qualitative biological knowledge is mathematically rigorously represented as a Boolean model, that is then converted into a continuous model. This continuous model can be used to explain quantitative experimental results and to design and optimize further experiments; the results of these experiments, in turn, help to refine the model.

Large-scale models of regulatory or signaling networks are often so-called *Boolean models *[1]. In fact, these models can be seen as the mathematically rigorous representation of qualitative biological knowledge. Their components, henceforth called *species*, can have only discrete states, typically two; these may be referred to as 0 and 1, 'off' and 'on', 'deactivated' and 'activated', etc. Time is discretized and the state of a species at time *t *+ 1 is a function of the states of the species at time *t*. Although being a crude simplification of biological reality, Boolean models are often able to reproduce the qualitative behavior of a system [2-8]. Naturally, Boolean models can neither describe continuous concentration levels nor realistic time scales. For this reason, they cannot be used to explain and predict the outcome of biological experiments that yield quantitative data. However, with increasing emphasis on these quantitative experiments the need for precisely this kind of model arises. In this contribution, we present and exemplify a practicable solution to this problem: a standardized method for accurately converting any Boolean model into a continuous model. This transformation fills the gap in the modeling process shown in Figure 1. It allows construction of a continuous model from qualitative knowledge by representing this knowledge as a Boolean model and then transforming this discrete model into a continuous one. The continuous model can now be used to explain experimental results and to design and optimize further experiments. The results of these experiments, in turn, help to refine the model.

Boolean models are a very coarse description of biochemical processes. They phenomenologically describe observed dependencies often leaving out still unknown players or intermediate steps. As our transformation requires no additional information the resulting continuous models are, of course, still phenomenological models. We can automatically create these continuous phenomenological models out of a Boolean model, but we cannot create a mass action law without additional knowledge on the biochemistry. Our method is a top-down approach for (large) networks with incomplete mechanistic knowledge — derived e.g. from pathway databases — where predictive kinetic modeling is infeasible. The main point that we want to make in this contribution is that also these phenomenological models can be used in Systems Biology to explain and predict quantitative experimental results.

**Figure 2:**
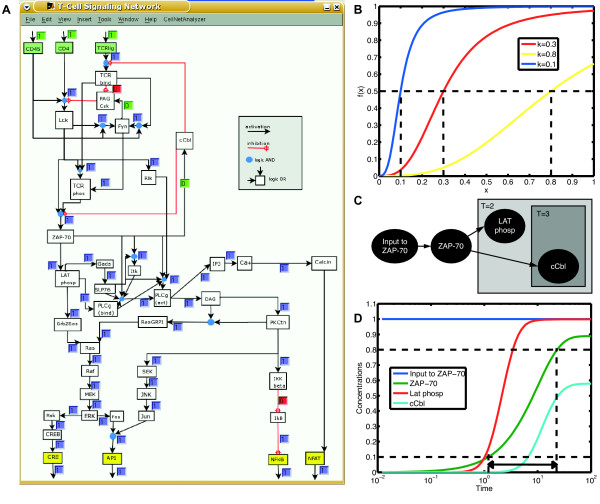
**T-cell model**. **(A) **Structure of the Boolean model as shown in *CellNetAnalyzer *[24]. **(B) **Hill functions with parameter *n *= 3 and different thresholds *k *= 0.3, *k*_fast _= 0.1 and *k*_slow _= 0.8. **(C) **Subnet of the T-cell model for scenario 2 (only activation) and scenario 3 (activation and feedback). **(D) **Numeric simulation of the subnet from (C). Time is plotted on a log-scale. The two dotted vertical lines indicate the time points when the concentration of ZAP-70 exceeds *k*_fast _and *k*_slow_, respectively.

To this end, we focus on a large Boolean model of T-cell activation proposed by Klamt et al. [6]. These cells play a pivotal role in the immune system. When foreign antigens bind to their receptors, signaling cascades are triggered within the T-cell leading to an activation of several transcription factors. The logical structure of the model is shown in Figure 2A. There are three inputs: the T-cell receptor TCR, the coreceptor CD4 and an input for CD45; as well as four outputs: the transcription factors CRE, AP1, NFkB and NFAT. The rephosphorylation of PAG-Csk by Fyn and cCbl mediated degradation are known to be slow processes compared to the other interactions. This is modeled by activating the feedback loops Fyn → PAG-Csk and ZAP-70 → cCbl only at a later stage. Therefore three scenarios are defined:

**scenario 1, resting state: **All inputs are set to 0, feedback loops are deactivated.

**scenario 2, early events: **Inputs are set to 1, feedback loops are still deactivated.

**scenario 3, mid-time events: **Inputs are still 1, feedback loops are active.

A qualitative analysis of an expanded version of this Boolean model by Saez-Rodriguez et al. [8] yielded new and non-obvious signaling pathways. There are also quantitative models covering aspects of T-cell signaling in mechanistic detail, e.g. [9]. In contrast thereto, the continuous model we obtain in the following describes the T-cell signaling cascade on a larger scale yet in less mechanistic detail.

We apply the transformation method to the T-cell model. The resulting continuous model is able to fully reproduce the behavior of the Boolean model. Moreover, we show that it can easily include and deal with the different time scales of interactions in the three scenarios. Hence the continuous model is indeed a generalization of the Boolean model with richer dynamic properties. This is in line with previous findings [10,11], indicating that the qualitative behavior of a discrete model is reproduced by its continuous homologue. We can further corroborate this hypothesis by generalizing existing theoretical results on the steady-states of discrete and continuous models.

The crucial question is, of course, whether the continuous model derived from a Boolean network is indeed competent to explain an aspect of biological reality in a precise quantitative fashion. We answer this question in the affirmative by showing that our T-cell model reproduces time courses of concentration levels measured for three different ligand concentrations [12]. Moreover, it is able to predict binding affinities of different ligands from their induced signaling profiles. The fact that the model can differentiate between more than two different concentrations shows that we have definitely left the Boolean (binary) world behind.

## Results

### Representation of Boolean functions and models

A *Boolean model *consists of

• *N *species *X*_1_, *X*_2_, ...., *X*_*N*_, e.g. genes, proteins, etc., each represented by a variable *x*_*i *_taking values in {0, 1},

• for each species *X*_*i *_a set of species  that influence *x*_*i *_and

• for each species *X*_*i *_an update function



giving the value of *x*_*i *_at the next time step for every possible combination of .

In the following we think of *B*_*i *_as a function on the vertices of the unit cube. Since vertex  corresponds to the AND gate



we can write *B*_*i *_in the form



This is a so-called *sum-of-product representation *of *B*_*i*_. These representations are especially convenient, as they allow to graphically represent our models using *interaction hypergraphs *[6]. The idea is to represent each product (AND gate) in the sum-of-product form of *B*_*i *_by a directed hyperedge between a set of start nodes  and the end node *X*_*i*_. Each pair (*s*, *X*_*i*_), *s *∈ *S*, carries a sign — '+' or '-' — depending on whether there is a factor *s *or ¬*s *in the product. All incoming hyperedges at node *X*_*i *_are then a graphical representation of *B*_*i*_. This is further illustrated in Additional data file 1.

### The general approach to making discrete models continuous

The first step for obtaining a continuous model from a Boolean one is to replace the discrete variables *x*_*i *_by continuous variables  taking values in [0, 1], i.e. we normalize concentrations to the unit interval. Consequently, we have to 'extend' the functions *B*_*i *_to functions : [0, 1]^*N *^→ [0, 1]. We call the functions *continuous homologues *of the Boolean functions *B*_*i*_. The crucial point is, how the transformation of a Boolean function into a continuous homologue is performed. We will address this issue in the next section and continue with the outline of the general approach.

In the second step we have to specify how to build the actual continuous model, for which there are two possibilities. The most straightforward is probably to proceed analogously to the Boolean model, i.e. to use discrete time steps and to compute the value of  at time *t *+ 1 by

(1)

In numeric simulations the discretization of time is obviously irrelevant. It complicates, however, the detection of small-scale continuous effects and is a serious drawback in the further investigation of the model by analytical methods.

Another way to build the continuous model is to try to mimic biological reality more closely: mRNAs, proteins, etc. are produced at a certain rate and are at the same time degraded. We assume the production of *X*_*i *_to be given by , and the degradation to be proportional to . Then the development of  over time is governed by the ordinary differential equation (ODE)

(2)

where *τ*_*i *_can be interpreted as the life-time of species *X*_*i*_. Note that due to the normalization of concentrations to the unit interval we have only one parameter for production as well as decay. To clarify this, assume that  denotes the non-normalized concentrations and  the corresponding production functions. Then a general system of ODEs is of the form , where *α*_*i *_is the production rate and *γ*_*i *_the decay rate of species *X*_*i*_. Since the maximal concentration of *X*_*i *_is *α*_*i*_/*γ*_*i *_we have the relation . It now follows that



and by setting *τ*_*i *_= 1/*γ*_*i *_and  we obtain the ODEs (2).

Herein we focus on model (2), as this model can be further analyzed using the rich and mathematically rigorous theory of ODEs. Note furthermore that model (1) can be considered a special case of model (2) after numeric integration.

### Continuous homologues of Boolean functions

As already mentioned, the key point is how a continuous homologue  can be obtained from a Boolean function *B*_*i *_in a computationally efficient manner. A suitable transformation has to satisfy three conditions:

**Accuracy: **It has to be accurate, which means that  and *B*_*i *_must agree on the vertices of the unit cube, i.e. for values in . Functions  satisfying this condition are called *perfect *continuous homologues. As will be shown, for perfect continuous homologues discrete and continuous models exhibit a similar steady-state behavior.

**Good analytical properties: **The functions  should have good analytical properties such as smoothness in order to allow and facilitate a mathematical analysis of the system of ODEs.

**Minimality and uniqueness: **The functions  should be the unique minimal solution in their interpolation class.

The three transformations we propose in the following are all based on multivariate polynomial interpolation [13,14] (see Methods). Here  is defined as a polynomial in the variables  that agrees with *B*_*i *_on the vertices of the unit cube. As will be shown in the Methods section, this technique satisfies all three of the above requirements. There are other approaches which we shortly review and compare in the Discussion section.

#### BooleCube

In a first step, we define the functions

(3)

by linearly interpolating the functions *B*_*i *_using the technique of multivariate polynomial interpolation as explained in the Methods section. These functions are called *BooleCubes*. By substituting  for *B*_*i *_in equation (2), we can then define a system of ODEs that describes the temporal development of the .

#### HillCube

The functions  are affine multilinear, i.e. for each 1 ≤ *j *≤ *N*_*i *_and fixed , *k *≠ *j*, there exist constants *a*, *b *∈ ℝ such that . Molecular interactions, however, are known to show a switch-like behavior, which can be modeled using sigmoid shaped *Hill functions *[15] (see Additional data file 2). The two parameters *n *and *k *have a clear biological meaning. The Hill coefficient *n *determines the slope of the curve and is a measure of the cooperativity of the interaction. The parameter *k *corresponds to the threshold in the Boolean model, above which one defines the state of a species as 'on'. Mathematically speaking, it is the value at which the activation is half maximal.

We now define a Hill function *f*_*ij *_with parameters *n*_*ij *_and *k*_*ij *_for every interaction and define new functions

(4)

which we call *HillCubes*. Plots of the HillCubes of all 16 two-variable Boolean gates can be found in Additional data file 3. Now a new system of ODEs can be defined by replacing the  in equation (2) by the HillCubes .

### normalized HillCube

Note that Hill functions never assume the value 1, but approach it asymptotically. Hence, the HillCubes are not perfect homologues of the Boolean update functions *B*_*i*_. If this is desired a simple solution is to normalize the Hill functions to the unit interval. This yields another (perfect) continuous homologue of the

Boolean functions *B*_*i*_

(5)

which we call *normalized HillCubes*, and thus defines also a new continuous ODE model.

### Theoretical results about the relation between discrete and continuous models

A natural and interesting question is how similar the discrete and the continuous model are. It can easily be shown (see Methods and [11]) that, whenever the  in equation (2) are perfect homologues of the Boolean update functions *B*_*i*_, a steady-state of the Boolean model will also be a steady-state of the continuous system. This result can be applied to the BooleCube model as well as the normalized HillCube model, but not to the non-normalized HillCube model. Therefore, Boolean steady-states will in general not be steady-states of the non-normalized HillCube system. The question is if Boolean steady-states still correspond to 'similar' steady-states of this continuous model, at least for certain parameters. Using the implicit function theorem we were able to show that this is indeed the case (see Methods). The reverse statement is, of course, not true, as in the continuous model additional (stable) steady-states may arise. Besides the steady-state behavior, monotony properties are a further important characteristic of a dynamical system. These properties determine the effect of a down- or up-regulation of a certain species on the other species. Due to its accuracy the presented transformation method preserves the monotony properties of a Boolean network. In the Discussion section we illustrate this further using the T-cell model as an example.

We show these results as they justify our transformation approach by demonstrating that it preserves essential properties of the Boolean model. For a deeper mathematical investigation of the relation between discrete and continuous models we refer the interested reader to the rich literature on this subject, e.g. [11,16,17]

### Simulation of the Boolean T-cell model

**Figure 3:**
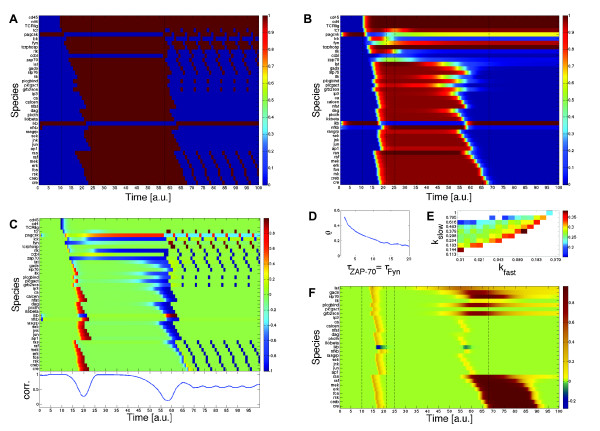
**Results from the Boolean and continuous simulations of the T-cell model using the manually determined parameter set**. The vertical dashed lines in (A, B, F) mark (from left to right): the switching on of the inputs, the total activation of the transcription factors, the activation of the feedback loops and the total deactivation of the transcription factors. **(A) **Boolean simulation using synchronous updates. **(B) **Continuous simulation using HillCubes. **(C) **Upper figure: difference between the time course from (A) and the time course from (B). Lower figure: correlation between the discrete and the continuous expression pattern of the lower 29 species at each time point 0 <*t *< 100. The curve was smoothened using a moving-average filter with 5 time-units window length. **(D) **Ratio of activation and deactivation time, *ρ*, for *τ*_Fyn _= *τ*_ZAP-70 _= 1, 2, ..., 20. Other parameters are set to *n *= 3, *k *= 0.3, *τ *= 1, *k*_fast _= 0.1 and *k*_slow _= 0.8. For *τ*_Fyn _= *τ*_ZAP-70 _= 1 the cascade was not activated properly. **(E) **Ratio of activation and deactivation time, *ρ*, for 20 different *k*_fast _and *k*_slow_. log10 (*k*_fast_) and log_10 _(*k*_slow_) are uniform from [-2, 0]. Other parameters are set to *n *= 3, *k *= 0.3, *τ *= 1, *τ*_Fyn _= *τ*_ZAP-70 _= 10. In the white areas the cascade was not activated properly. **(F) **Comparison between time course *y*(*t*) with the manually determined parameters and time course *y*^3^(*t*) with the decreased threshold in the activation of LAT-phosp by ZAP-70 as described in the section about monotony properties. Plotted is the difference *y'*(*t*) - *y*(*t*).

Figure 3A shows a simulation of the Boolean T-cell model using synchronous updates. In the beginning the three inputs were 0 and the system was in its resting state (scenario 1). At *t *= 10 the inputs were manually switched on and the signaling cascade was triggered off, which at *t *= 24 led to the activation of all four transcription factors CRE, AP1, NFkB and NFAT (scenario 2). At *t *= 57 we activated the feedback loops Fyn → PAG-Csk and ZAP-70 → cCbl. Consequently the cascade was blocked and at *t *= 67 all transcription factors were again deactivated. So, essentially the simulation used three different Boolean models at times [0, 10), [10, 57) and [57, 100]. Note that in the Boolean model the time point for the activation of the feedback loops could be chosen arbitrarily. As is explained below, in the continuous model this time point was determined by our choice of the kinetic parameters. We chose *t *= 57 since then deactivation of all transcription factors occurred at around the same time point in both models.

After deactivation oscillations occurred, artefacts of the synchronous updating. When the species were updated asynchronously according to some permutation, these oscillations could be observed for 3078 out of 10000 randomly sampled permutations; in the other cases a steady-state was reached.

### Continuous T-cell model

We applied the transformation technique to the Boolean T-cell model. Using non-normalized HillCubes we obtained a large quantitative model of T-cell activation (see Methods). In a first step, we manually determined approximate parameters for which the continuous model reproduces the behavior of the Boolean model during all three scenarios. The information about fast and slow interactions was thereby encoded in the values of specific parameters. Consequently, the continuous model was able to explain both, the activation as well as the deactivation of T-cells, without any alteration of the network topology between different scenarios. Note that this was necessary in the Boolean case. We set all Hill coefficients to *n *= 3, the thresholds to *k *= 0.3 and the life-times to *τ *= 1, with the exception of Fyn and ZAP-70. Here we knew that the interactions Fyn → PAG-Csk and ZAP-70 → cCbl operate on a slower time scale. Therefore the thresholds for these interactions were set to *k*_slow _= 0.8 whereas the thresholds for the interactions Fyn → TCR-phos, ZAP-70 → LAT-phosp, ZAP-70 → PLCg (act) and ZAP-70 → Itk were set to *k*_fast _= 0.1. The Hill functions for the three thresholds *k*, *k*_slow _and *k*_fast _are displayed in Figure 2B. Finally, we set the life-times of Fyn and ZAP-70 to *τ*_Fyn _= *τ*_ZAP-70 _= 10 to enlarge the time gap between the two switching points.

The numeric simulation of the continuous T-cell model with the manually determined parameters is shown in Figure 3B. At first, the cell was again in its resting state with all three inputs turned off. Then at *t *= 10 we manually switched on all inputs and the signaling cascade was triggered off showing an expression profile very similar to the Boolean simulation (Figure 3A). We observed a total activation of the four transcription factors at around *t *= 21 just like in the Boolean simulation. One can clearly see that Fyn and ZAP-70 were activated more slowly. Nonetheless TCR-phos, LAT-phosp, PLCg (act) and Itk were instantly turned on due to the low threshold *k*_fast_. Only when at around *t *= 25 the concentrations of Fyn and ZAP-70 were high enough, the feedback loops Fyn → PAG-Csk and ZAP-70 → cCbl became active and began to switch on PAG-Csk and cCbl. While PAG-Csk reached a constant medium expression level, cCbl was only weakly and transiently expressed. This, however, sufficed to switch off the cascade and ultimately at around *t *= 67 all transcription factors were again deactivated. In contrast to the Boolean model, the continuous model did not exhibit an oscillatory behavior but reached a steady-state after deactivation of the signaling cascade. The species with long expression periods in the Boolean oscillation (Tcr bind, PAG Csk, Fyn, TCR phos and IkB) were expressed at high or medium levels whereas the species with short expression periods were not expressed at all.

### Comparison of the discrete and the continuous T-cell model

Although one can argue that there is no real time scale in Boolean networks, we compared both models by substracting the discrete from the continuous time course (Figure 3C). The activation of the feedback loops in the Boolean model was conveniently chosen such that we have a total deactivation of the transcription factors at around *t *= 67 in both models. While we observed an almost perfect agreement of the time courses of the three inputs, there was a huge difference in the time courses of the species involved in the two feedback loops. This was not surprising, considering that these loops are regulated differently in both models. The species downstream of the regulatory loops (from LAT phosp to CRE in Figure 3C) showed again a similar expression pattern.

We then analyzed this part of the cascade more deeply. Figure 3C also shows the correlation between the discrete and the continuous expression pattern of the species downstream of the feedback loops at each time point 0 <*t *< 100. We observed a high correlation in the more stationary phases (resting state, activated state and deactivated state) and a significant drop of correlation during the transitions between these phases. This met our expectation that the two models show the same qualitative but a different dynamic behavior.

### Ratio of activation and deactivation time

When looking at Figures 3A and 3B, a striking difference between the dynamics of both models is that in the discrete model activation and deactivation took approximately the same time, whereas in the continuous model activation was a much faster process than deactivation. This can also be seen from the red and blue 'steps' in upper Figure 3C during the activation and deactivation phases. To confirm that this was not merely an artefact of our choice of parameters, we calculated and analyzed the ratio between activation and deactivation time for different parameters. We defined

• the beginning of activation *t*_ActBeg _as the time when the first species (of the lower 29) reaches 5% of its maximal value,

• the end of activation *t*_ActEnd _as the time when the last species reaches 95% of its maximal value,

• the beginning of deactivation *t*_DeactBeg _as the time after *t*_ActEnd _when the first species drops under 95% of its maximal value,

• the end of deactivation *t*_DeactEnd _as the time when the last species drops under 5% of its maximal value,

• and, finally, the ratio of interest



In the Boolean model we could easily compute *ρ*_b _= 1 implying equally fast activation and deactivation. In the continuous model the crucial parameters were the life-times *τ*_Fyn _and *τ*_ZAP-70 _on the one hand and the concentration thresholds *k*_fast _and *k*_slow _on the other hand. The remaining parameters were set to *n *= 3, *k *= 0.3 and *τ *= 1.

First, we computed *ρ *for fixed *k*_fast _and *k*_slow _and different *τ*_Fyn _= *τ*_ZAP-70_. The result is shown in Figure 3D. For *τ*_Fyn _= *τ*_ZAP-70 _= 1 the cascade was not activated properly. For larger values we observed a decrease in *ρ *implying that an increase of *τ*_Fyn _and *τ*_ZAP-70 _prolonged the deactivation phase. This was to be expected — longer life-times resulted in a lessened increase of the decisive elements Fyn and ZAP-70 in the regulatory loop.

Second, we analyzed the effect of *k*_fast _and *k*_slow _for fixed *τ*_Fyn _= *τ*_ZAP-70 _= 10. The result is shown in Figure 3E. Only for parameters *k*_fast _≪ *k*_slow _< 1 the cascade was activated properly. This agrees well with the fact that the difference between these two parameters is responsible for the delayed activation of the feedback loops. If it was not big enough the cascade was being deactivated before it had been fully activated. The greater this difference was, i.e. the farther we go away from the diagonal in Figure 3E, the smaller *ρ *got, implying that a later activation of the feedback loops prolonged the deactivation phase. However, despite all these influences of the parameters, we observed much smaller ratios *ρ *in the continuous model than in the Boolean model. The average *ρ*'s in Figures 3D and 3E were  = 0.24 and  = 0.27, respectively, which were both significantly smaller then *ρ*_b _= 1. This suggests that *ρ *≪ 1 may be an invariant of the dynamical system of biological importance.

### Explanation of experimental data using the continuous T-cell model

**Figure 4:**
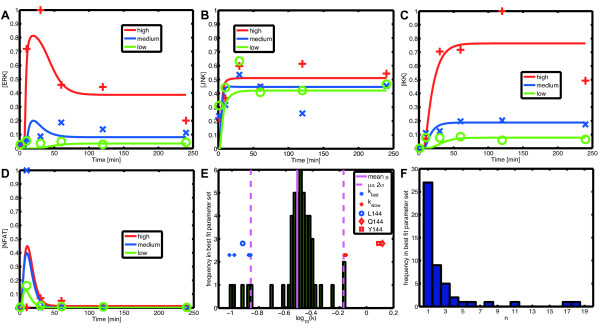
**Results of the parameter fit**. **(A-D) **Simulation of the continuous model (solid lines) for high (red), medium (blue) and low (green) concentrations of ligand L144 and experimentally measured concentrations, cf. markers '+', '×' and '○'. **(A) **ERK. **(B) **JNK. **(C) **NFAT. **(D) **IKK. **(E, F) **Distribution of the Hill parameters in the best fit parameter set for ligand L144 (Table 1). **(E) **Distribution of the thresholds *k*. The markers '+' indicate the position of the parameters which we set to *k*_fast _(blue) and *k*_slow _(red) at the beginning of the optimization. The marker '○' indicates the position of *k*_L144_; for comparability the thresholds *k*_Q144 _and *k*_Y144 _are also indicated, cf. the markers '◇' and '□', respectively. **(F) **Distribution of the Hill exponents *n*.

The crucial question is if our model can reproduce real data. To test this we used the data set presented in [12] (see Methods). It describes the dynamics of the activation of key signaling elements upon activation of the TCR by three different ligands with varying affinity, Q144, Y144 and L144. We considered, in particular, the ligand L144 for which experiments were performed with three different ligand concentrations. Using global minimization of the model fit with respect to the data, we were able to determine a set of parameters for which our model reasonably reproduced the experimental data (see Methods and Table 1). Figures 4A-D show the corresponding simulated time courses. We see that the model was able to approximate the time courses of ERK and IKK well. The fit of JNK was also acceptable, although the high measured concentrations at time *t *= 0 constituted a problem, as the model was naturally unable to reproduce them due to the delaying effect of the signaling cascade. In the case of NFAT, the model was unable to reproduce the non-monotone dependence on the ligand concentration. This suggests that the network structure cannot be reconciled with the non-monotone dependence of the two key signaling molecules JNK and especially NFAT with respect to the ligand concentration. A non-monotone response of T-cell signals has been reported in other contexts [18], and is consistent with the role of T-cells: their response has to be exquisitely regulated so as to reply only to a particular stimulus; an uncoupled response between the JNK (and p38) and ERK MAP Kinases has also been observed [19]. The regulation of NFAT and JNK, and more generally of TCR-induced signaling, is complex and not yet fully understood. To mention just two examples, processes such as Ras localization [20] have been shown to play a key role, but are not included in the model, and the regulation of calcium, which governs NFAT behavior, is more complex than described in the model. It is out of the scope of this paper to investigate this intriguing behavior in detail, but this result illustrates the power of our approach to gain new biological insight: taking exactly the same knowledge encoded in the discrete model and fitting it to quantitative data, we were able to identify the incompleteness of our model in an aspect that we could not have explored with a discrete model.

**Table 1:** Best fit parameter set for ligand L144.

**Species**	**Input**	***n***	***k***	**Input**	***n***	***k***	**Input**	***n***	***k***	***τ***
zap70	tcrphosp	2	0.29	lck	1	0.33	ccbl	1	0.30	2.77

tcrphosp	tcr	1	0.34	lck	1	0.34	fyn	1	0.13	0.03

tcr	TCRlig	1	0.20	ccbl	1	0.41				0.59

slp76	gads	1	0.35							0.24

sek	pkcth	1	0.26							0.01

rsk	erk	**3**	**0.30**							**1.00**

rlk	lck	1	0.36							1.02

rasgrp	pkcth	1	0.40	dag	1	0.39				2.76

ras	rasgrp	3	0.33	grb2sos	17	0.39				7.50

raf	ras	3	0.31							11.80

plcgbind	lat	5	0.36							0.01

plcgact	zap70	1	0.10	slp76	1	0.29	rlk	1	0.24	0.01
		
	plcgbind	1	0.31	itk	2	0.34				

pkcth	dag	2	0.31							8.66

pagcsk	tcr	1	0.32	fyn	1	0.69				1.33

nfkb	ikb	**3**	**0.30**							**1.00**

nfat	calcen	1	0.37							5.03

mek	raf	1	0.28							0.01

TCRlig	ext. sig.	1	0.12							1.28

lck	pagcsk	2	0.30	cd4	2	0.37	cd45	1	0.33	0.15

lat	zap70	3	0.14							0.01

jun	jnk	**3**	**0.30**							**1.00**

jnk	sek	4	0.57							0.08

itk	zap70	2	0.10	slp76	1	0.31				0.01

ip3	plcgact	2	0.38							0.01

ikkbeta	pkcth	18	0.33							10.55

ikb	ikkbeta	**3**	**0.30**							**1.00**

grb2sos	lat	8	0.46							5.26

gads	lat	1	0.36							0.12

fyn	tcr	6	0.27	lck	2	0.30	cd45	4	0.37	0.46

fos	erk	**3**	**0.30**							**1.00**

erk	mek	2	0.28							0.10

dag	plcgact	3	0.28							0.01

creb	rsk	**3**	**0.30**							**1.00**

cre	creb	**3**	**0.30**							**1.00**

cd4	ext. sig.	1	0.32							2.25

cd45	ext. sig.	1	0.35							1.64

ccbl	zap70	1	0.70							0.29

calcen	ca	11	0.30							4.53

ca	ip3	3	0.33							0.20

ap1	jun	**3**	**0.30**	fos	**3**	**0.30**				**1.00**

The bold entries are the parameters downstream of all measured species. They were not affected by the optimization process.

### Best fit parameter set

Subsequently, we analyzed the distribution of the Hill parameters within the best fit parameter set for ligand L144 (Table 1). When looking at the distribution of the exponents *n *upstream of the measured species (Figure 4F) we found that after the optimization 36 out of 49 (73%) were below the ad hoc estimate (3) and only 8(16%) were above it. This was to be expected, as in order to fit three different concentration levels the model had to contain mainly slow switches (low *n*) and only few Boolean-like switches (*n *→ ∞). Figure 4E shows the distribution of the threshold parameters *k *upstream of the measured species. As explained above, we had manually set these parameters to 0.3, with the exception of four *k*'s which had been set to *k*_slow _= 0.8 and two *k*'s which had been set to *k*_fast _= 0.1. Interestingly, this structure had been preserved during the optimization. The mean of the thresholds *k *was 0.298 and hence well agreed with the ad hoc estimate. Also the high and low thresholds were still at least two standard deviations away from the mean, cf. the red and blue markers '+' in Figure 4E. Only one other parameter *k *also had a *Z*-score above 2: the threshold *k*_L144 _for the stimulation of the TCR by the ligand L144, cf. marker 'o'. Possible implications hereof are discussed below.

Due to the large number of parameters the obtained parameter set was, of course, far from being unique. But we showed that a continuous model inferred from a Boolean model is able to reproduce experimental data in a quantitative way. Moreover, this transformation could enhance the explanatory power of the model in the sense that it was enabled to differentiate between more than two states.

In our example, the threshold parameters *k *are rather tightly centered around their mean. In principle, however, we could also have extreme outliers, i.e. very large or very small (≈ 0) values in the distribution. Mapped back to the Boolean model, this would imply a change of the network topology, as the corresponding reactions are then quasi-constant, either 'off' (for very large *k*) or 'on' (for very small *k*). Thus, a fitting of the continuous model to experimental data may also yield information about the network structure of the Boolean model.

### Prediction of binding affinities of different ligands

As already mentioned, the fitted threshold *k*_L144 _for the stimulation of the TCR by the ligand L144 was significantly below the mean of the other thresholds *k*, cf. marker 'o' in Figure 4E. This gave rise to the question of which affinities the model predicts for the other two ligands. To this end, we fitted the affinities of Q144 and Y144, mapped to the inverse of the Hill function's threshold parameters *k*_Q144 _and *k*_Y144_, respectively, keeping the rest of the parameters constant. As expected, the fits themselves were far from perfect, due to parameter indeterminacies. Surprisingly however, the values of *k*_Q144 _and *k*_Y144 _we obtained were significantly above the mean of the other thresholds *k*, cf. markers '◇' and '□' in Figure 4E. This suggests that the predicted relation between the parameters *k*_L144 _≪ *k*_Y144 _<*k*_Q144 _is not simply an artefact of the optimization process. And indeed, it agrees well with experimental data [21].

## Discussion

We now further discuss the presented transformation method and compare it to various other approaches. Also the relation between discrete and continuous models is discussed, especially with respect to their steady-state behavior and monotony properties.

### Comparison of different transformation approaches

The relation between discrete and continuous models has already been investigated and various approaches to the problem of constructing the continuous homologues of the Boolean update rules have been proposed. In the following we shortly review previous work and compare the different approaches.

#### Piecewise linear differential equations

The idea to compare continuous and discrete models is almost as old as Boolean modeling itself. In 1973, Glass et al. [11] studied the relation between discrete models and ODE models of the form (2). Their motivation, however, was quite the opposite of ours. While we intend to enrich the dynamic behavior of discrete models, Glass et al. wanted to investigate the qualitative properties of continuous networks by studying corresponding simpler discrete models. They propose Hill functions as a suitable continuous homologue of one-variable Boolean step functions. In the case of multi-variable Boolean functions *B*_*i *_a (perfect) continuous homologue  is constructed as follows: Note that, when building a Boolean model, one implicitly introduces a threshold 0 <*θ*_*i *_< 1 for each species *X*_*i *_and defines its state as 'on' if its concentration is above this threshold. The hyperplanes  = *θ*_*i*_, *i *= 1, 2, ..., *N *decompose the cubes  into  rectangular regions called *domains*. Each of these domains contains exactly one vertex  and is denoted by  accordingly (see Additional data file 4). A simple way of defining the functions  is now to set

(6)

With this definition model (2) is a so-called *piecewise linear ODE model *which means that within each domain  equation (2) is a linear ODE of the form . This kind of equation is very well understood and can be solved analytically. The functions  defined in (6) perfectly agree with *B*_*i *_on the vertices of the unit cube. Using piecewise linear ODEs Glass et al. could prove some theoretical results on the relation between discrete and continuous models, e.g. that Boolean steady-states are also steady-states of the continuous model. Some of these results are restated and generalized in the Methods section. Piecewise linear models are typically not used for quantitative simulations, as the step-like transition between the different domains is often unrealistic. Rather they are analyzed in a qualitative and semi-qualitative way, where their trajectories between the different domains are treated analogously to the state transition graphs of Boolean models [22].

#### Fuzzy logic

Another well studied way of generalizing Boolean models is *fuzzy logic *[23]. Recall that in a Boolean model one defines the state of a species as 'on' if its concentration is above a certain threshold. In fuzzy logic this concept is relaxed and a so-called *degree of membership *(DOM) function  is introduced for each species *X*_*i*_. For concentrations 0 =  = 1 this function gives the degree with which we say that *X*_*i *_is 'on'. There are two standard ways of generalizing the Boolean operators AND, OR, NOT:

(i) min-max logic

(7)

(ii) product-sum logic

(8)

**Figure 5:**
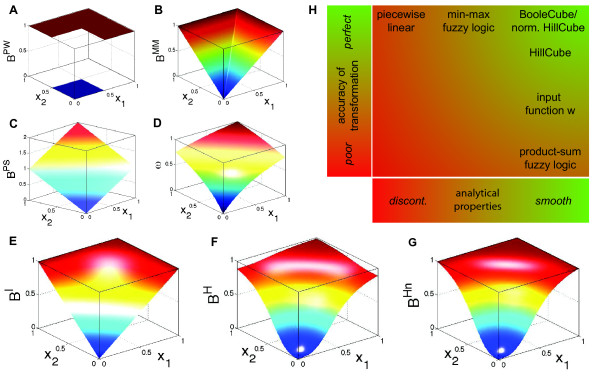
**Comparison of different transformation techniques**. Different continuous homologues of a Boolean OR gate. **(A) **Piecewise linear function . **(B) **Function  obtained by min-max fuzzy logic and linear DOM functions. **(C) **Function  obtained by product-sum fuzzy logic and linear DOM functions. **(D) **Input function *ω *introduced by Mendoza et al. [10]. **(E) **BooleCube  obtained by multivariate polynomial interpolation. **(F) **HillCube . **(G) **normalized HillCube . In the last two figures parameters *n *= 3 and *k *= 0.5 were chosen for both inputs. **(H) **Overview of the different transformation techniques with respect to their analytical properties and transformation accuracy.

When using product-sum logic, a normalization to the unit interval is necessary, since  can assume values greater than 1. Both, (i) as well as (ii) are ways to construct the functions  from the Boolean functions *B*_*i*_. If the DOM functions satisfy *μ*(0) = 0 and *μ*(1) = 1, the functions  obtained by min-max logic agree with *B*_*i *_on the vertices of the unit cube and hence the steady-states of the Boolean model are also steady-states of the continuous model. The major drawback of this method is that the functions  are in general not differentiable, and do not have a 'nice' analytic representation (Figure 5B). Hence most analysis methods are not applicable to the resulting ODE systems. When we use product-sum logic, we encounter the problem that the resulting functions  do not necessarily agree with *B*_*i *_(Figure 5C).

#### Standardized qualitative dynamical systems

Mendoza et al. [10] put forward a method to transform a Boolean model into a system of ODEs similar to (2) called *standardized qualitative dynamical system*. Their approach, however, is applicable only to a subclass of Boolean models: For each species *X*_*i *_we have a set of activators *A*_*i *_and a set of inhibitors *I*_*i *_of *X*_*i*_. Then *x*_*i *_is set to 1 at the next time step if at this time any of its activators and none of its inhibitors are acting upon it, otherwise *x*_*i *_is set to 0. This corresponds to the Boolean logic . Clearly, many logic functions, like XOR gates for example, cannot be represented that way. From this Boolean model a continuous model is built up consisting of ODEs of the form



Where

(9)

The right-hand side of the above ODE consists of two parts: an activation function and a term for decay as in (2). The activation is given by a sigmoid shaped function of *ω*_*i*_, where *ω*_*i *_represents the total input to node *X*_*i*_. The steepness of the activation function is determined by the parameter *h*. Decay is assumed to be proportional to . Actually, a more general form of *ω*_*i *_is introduced in [10], where the influence of the activators and inhibitors can be differently weighted. For the sake of better comparability we set all these weights equally to 1, as suggested in [10]. The more activators and the less inhibitors of a node are 1, the greater *ω*_*i *_is. It takes its minimum (0), iff all activators are 0 or all inhibitors are 1, and its maximum (1), iff all activators are 1 and all inhibitors are 0. This, however, does not exactly correspond to the Boolean rule from above. In consequence, steady-states of the Boolean model are not necessarily steady-states of the continuous model. This will be further discussed below.

#### Multivariate polynomial interpolation

The aforementioned attempts only lead to functions which are either not differentiable or do not precisely generalize the Boolean logic. A straightforward approach to eliminate these drawbacks is to use multivariate polynomial interpolation [13] for the construction of the functions  (see Methods). This technique can be applied to any Boolean function *B*_*i*_. The resulting BooleCubes  are smooth and can easily be analytically differentiated and integrated. They agree with the Boolean functions *B*_*i *_on the vertices of the unit cube and hence the steady-states from the Boolean model are also steady-states in the continuous model.

**Figure 6:**
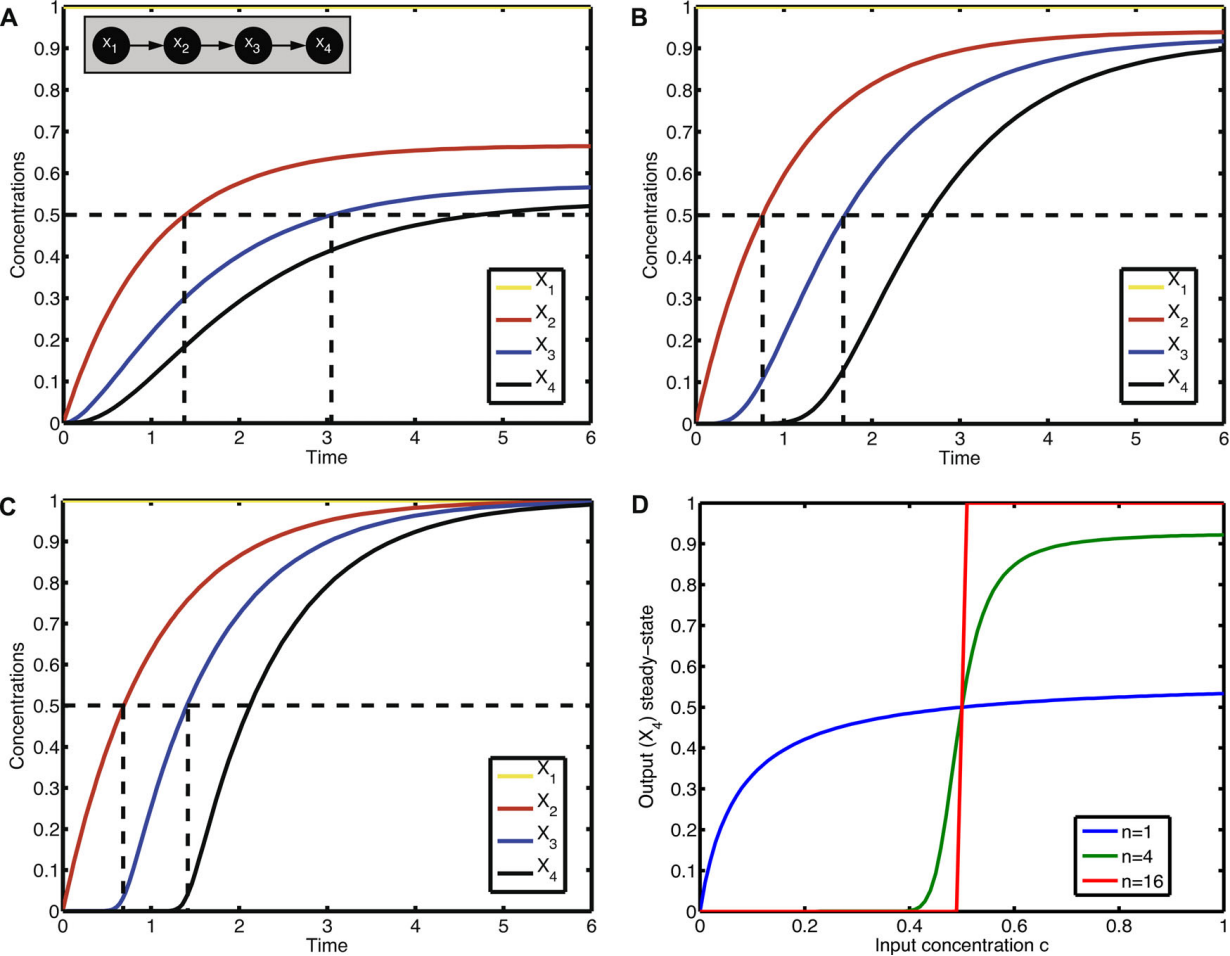
**Effect of increasing Hill exponents**. We consider a simple cascade between the four species *X*_1_, *X*_2_, *X*_3_, *X*_4 _as shown in the inset in **(A)**. Each activation is modeled using a Hill function with threshold *k *= 0.5 and Hill coefficient *n*. The life-times *τ*_*i *_are set to 1. As initial conditions we take  = *c *> 0,  = 0,  = 0,  = 0, for some constant input concentration *c*. The input node *X*_1 _remains constant and the other concentrations  change accordingly to the ODE , *i *= 2, 3, 4. We simulate the model for different Hill coefficients *n *= 1, 4, 16 and input level *c *= 1; the results are shown in **(A)**, **(B) **and **(C)**. All three time courses show qualitatively the same cascade-like pattern. With growing *n*, however, the onset of activation of *X*_3 _and *X*_4 _comes closer and closer to the time point at which their activators *X*_2 _and *X*_3_, respectively, cross the threshold *k*. **(D) **shows the input-output curve. Plotted is the (constant) input concentration *c *of node *X*_1 _against the steady-state concentration of node *X*_4_. For *n *> 1, we observe the typical sigmoid stimulus-response behavior of signaling cascades, see e.g. [28]. With increasing *n *the steepness of the input-output curve increases, leading to an almost discrete (Boolean) output in the case *n *= 16.

We define Hill functions *f*_*ij *_for all interactions and consider the HillCubes  from equation (4). The idea behind this is that each interaction is described by its own Hill function with specific parameters and the different interactions are coupled by the BooleCubes. Since they are affine multilinear, the latter preserve the shape of the individual Hill functions. Thus, we can mimic single-component non-linearities which are common in switch-like regulatory systems. Additional data file 3 shows the HillCubes derived from all 16 two-variable Boolean gates. A mathematically rigorous treatment of this kind of dynamical systems can be found in [14]. If the Hill coefficient *n *goes to infinity the Hill function becomes more and more like a Boolean step function (see Additional data file 2). Hence for large exponents the HillCubes are very similar to the Boolean functions *B*_*i *_and the continuous system will likely show an almost Boolean behavior. This is further illustrated in Figure 6.

The HillCubes do not perfectly agree with the Boolean update functions due to the asymptotic behavior of the Hill functions. By a suitable choice of the Hill parameters the difference can be reduced but not fully eliminated. An easy way to achieve a perfect agreement is to normalize the Hill functions to the unit interval, as is done in the normalized HillCubes  from equation (5).

#### Comparison

To conclude, we illustrate the above methods applied to a simple OR gate between two species *X*_1 _and *X*_2_. We compute

• the piecewise linear function



from equation (6),

•  obtained by fuzzy logic (with linear DOM functions) following (7),

•  obtained by fuzzy logic (with linear DOM functions) following (8),

• the input function from equation (9) introduced by Mendoza et al.



• the BooleCube  from equation (3) obtained by the interpolation technique,

• the HillCube  from equation (4) for Hill functions *f*_1 _and *f*_2 _with parameters *n *= 3, *k *= 0.5,

• and, finally, the normalization



of  from equation (5).

Figures 5C and 5D show the product-sum fuzzy logic function  and the input function *ω*. One can clearly see that they do not represent a pure OR gate, where the values at (*x*_1_, *x*_2_) = (1, 0) and (*x*_1_, *x*_2_) = (0, 1) should already be maximal. This is the case in Figures 5A and 5B which show the piecewise linear  and the min-max fuzzy logic function . Here however, the problem is that the functions are not differentiable, as can easily be seen from their plots. The BooleCube  shown in Figure 5E is both, smooth and maximal as soon as any concentration is equal to 1. Finally, Figures 5F and 5G show the (normalized) HillCubes  and , respectively, which are also smooth and can be considered good transformations of the Boolean OR gate. An overview about the discussed advantages and disadvantages of the different transformation techniques is provided in Figure 5H.

### Theoretical results about steady-states

A fundamental principle of biological modeling is that steady-states of a model typically correspond to the different operating modes or states of the biological system under study. This correspondence was also the motivation for Kauffman's seminal study [1], where Boolean models were introduced for the first time in biology. A critical step in the justification of any transformation method therefore is to ensure that at least the steady-states of the Boolean model are still steady-states in the homologue continuous system. In the case of a perfect agreement of the Boolean update rules *B*_*i *_with their continuous homologues  this can easily be shown (see Methods and [11]). This perfect agreement, however, is not a biologically plausible assumption; biological interactions, such as enzyme kinetics for example, are known to asymptotically approach but never fully reach a saturation level. Empiric evidence that also in real-world examples, the steady-states of a Boolean model correspond to steady-states of a homologue continuous model, is given by Mendoza et al. [10]. The method of transformation used therein has already been described above and we also mentioned that it does not accurately transform the Boolean update rule into a continuous activation function. This inaccuracy is due to a systematic difference between the Boolean logic and the analytic form of the activation function. It is not the result of an asymptotic sigmoid function; in fact, the used sigmoid function assumes its maximal values 0 and 1. One can easily construct an example where due to this systematic difference Boolean steady-states are not conserved under the transformation (see Additional data file 5).

In the case of the HillCube model, it is the other way round. There is no systematic difference between the Boolean update rules and the HillCube functions, the imperfect agreement is caused by the asymptotic behavior of the Hill functions. Therefore, the difference between both can be made arbitrarily small — albeit not zero — by a suitable choice of parameters. In this situation, we can show that for certain parameters, more precisely for sufficiently large exponents, there will be a steady-state of the continuous system in the neighborhood of each Boolean steady-state (see Methods). This theoretical result further justifies the presented transformation method.

### Monotony properties

A nice feature of our method for converting Boolean into continuous models is that monotony properties, typically captured in the underlying interaction graph of the system, are preserved. Interaction graphs are signed directed graphs where each directed edge reflects a causal dependency, which can either be positive or negative, between its start and end node. Boolean models represented as interaction hypergraphs have a unique underlying interaction graph which can easily be derived from the logical model (by splitting the ANDs, see Klamt et al. [6]). For example, the Boolean function *A *= (¬*B *∧ *C*) ∨ *D *would be translated into two positive arcs (*C *→ *A*, *D *→ *A*) and one negative arc (*B *⊣ *A*). In the interaction graph one may then compute the recently introduced dependency matrix [6,24], which determines for each pair (X, Y) of species the global effect of X on Y. This effect can — in some cases only initially — be positive, negative, ambivalent or vanishing.

For example, the dependency matrix of the T-cell model tells us, that LAT-phosp exerts purely positive effects on ERK, JNK, IKK, and NFAT, because there are only positive paths from LAT-phosp to these species and no negative feedback is involved. If we simulate a scenario with the logical model and repeat it then with e.g. fixing LAT-phosp to 1 (i.e. to the highest possible value), the resulting Boolean values in the four species mentioned above cannot decrease. In continuous systems, the interaction graph is encoded in the sign structure of the Jacobian matrix. In fact, in a continuous system obtained from a Boolean model the interaction graph is up to negative self-loops identical and monotony properties are therefore preserved. Accordingly, a positive perturbation in LAT-phosp, e.g. by permanently decreasing the threshold of ZAP-70 in the interaction activating LAT-phosp, results in a trajectory that is always above the trajectory of the non-perturbed system (Figure 3F). In fact, in accordance with the interaction graph of the Boolean model, we observe purely positive effects on all species downstream of LAT-phosp with the exception of ikB. Hence, important qualitative properties of the dynamics derived from the logical model are reflected in the dynamics of the continuous system.

## Conclusion

With increasing amounts of quantitative data being available, the challenge arises how we can use our typically qualitative knowledge about biological systems to explain this data. For this purpose, we presented a canonical and fully standardized way of transforming qualitative discrete into continuous models. The transformation is accurate and we can show that it preserves the steady-state behavior as well as the monotony properties of the discrete model. The feasibility of the presented approach was substantiated by applying it to a logical model of T-cell receptor signaling. The resulting model is an extensive continuous model of T-cell activation. In contrast to the Boolean model it allowed to accommodate different time scales by adjusting kinetic parameters. It was competent to reproduce time courses of key signaling molecules measured for three different ligand concentration levels. Moreover, the model was able to predict the binding affinities of different ligands.

Being fully automatized [Krumsiek et al.: Odefy — From discrete to continuous models. In preparation (2009)] the presented method recommends itself to be applied to further biological systems. Future work could also aim at generalizing the approach from Boolean (binary) to *s*-state systems, where one no longer differentiates between two but *s *> 2 discrete states, e.g. 'low', 'medium' and 'high'. Finally, the relation between a discrete model and its continuous homologue needs to be further investigated, especially with respect to more complex behaviors like oscillations, which are of importance in many biological systems, such as cell cycle.

## Methods

### Multivariate polynomial interpolation

We now explain the technique of multivariate polynomial interpolation of a single Boolean function *B*_*i*_. Therefore *i *∈ {1, 2, ..., *N*} is fixed and for the sake of simplicity the subscript *i *is omitted. We remark that here *B *can be any real-valued function on the vertices of the unit cube {0, 1}^*N*^, i.e. does not necessarily have to be a Boolean function. The idea is, to find a polynomial : ℝ^*N *^→ ℝ that is a continuation of *B*_*i *_in the sense that  for all .

One can easily see, that there is no unique solution to this problem. Therefore, we additionally require that the degree of the polynomial be minimal, where the degree of some polynomial



 for almost all (*m*_1_, *m*_2_, ..., *m*_*N*_) ∈ ℕ^*N*^, is defined as



For  we define



We now show that  indeed satisfies the three requirements for interpolation functions that we set out at the beginning (see section on continuous homologues of Boolean functions).

**Theorem. ***The function **has the following properties:*

*(i) **for all *.

*(ii) **is the unique minimal degree polynomial interpolating B.*

*(iii) Let s denote the number of symmetry hyperplanes of B, i.e. the number of variables x*_*i *_*satisfying*

*B*(*x*_1_, ..., *x*_*i*-1_, 0, *x*_*i*+1_, ..., *x*_*N*_) = *B*(*x*_1_, ..., *x*_*i*-1_, 1, *x*_*i*+1_, ..., *x*_*N*_), *for all *(*x*_1_, ..., , ..., *x*_*N*_) *ε *{0, 1}^*N*-1^. *Then *deg () = *N *- *s*.

*(iv) **is affine multilinear. It is multilinear iff it corresponds to an AND gate, i.e.*

*B*(*x*_1_, *x*_2_, ..., *x*_*N*_) = .

*(v) Let *. *Then it holds*



*In particular*, *if B is a Boolean function.*

*Proof. *(i) Note that for  ∈ {0, 1} we have



(ii) A minimal degree interpolation polynomial is of the form



since exponents greater than 1 can be replaced by 1 without changing the values of *f *for . We order the vertices of the unit cube such that the number of 1's in the coordinates is not decreasing and denote the reordered sequence by . Then we consider the sequence of equations *f*(*V*_*k*_) = *B*(*V*_*k*_), *k *= 1, 2, ..., 2^*N *^. For all *k *∈ {1, 2, ..., 2^*N*^} there is exactly one coefficient  whose monomial  satisfies *g*(*V*_*k*_) = 0, *k *= 1, 2, ..., *k *- 1 and *g*(*V*_*k*_) = 1. This allows to uniquely determine the coefficients  and that way also the polynomial *f*. Since  is of the form (*), it is the unique minimal degree interpolation polynomial.

(iii) The degree of  is clearly deg () ≤ *N*. If *B *is symmetric with respect to the hyperplane *x*_*i *_= 0.5, w.l.o.g. *i *= 1, we have *B*(0, *x*_2_, ..., *x*_*N*_) = *B*(1, *x*_2_, ..., *x*_*N*_) for all (*x*_2_, ..., *x*_*N*_) ∈ {0, 1}^*N*-1 ^and consequently



Hence, deg () ≤ *N *- 1. Inductively this proves (iii).

(iv) Let *i *∈ {1, 2, ..., *N*}. Then at fixed  the derivative  is constant so  is affine linear in . Moreover, we have



For the last equivalence note that if  is multilinear, then  if any  = 0.

(v) Assume  and w.l.o.g. . Then it follows from (iv) that  or . Inductively, we obtain *x *∈ {0, 1}^*N *^such that , a contradiction to (i). Analogously, one proves that .   □

Note that in general deg () = *N *- *s *does not hold, as can be seen from the Boolean function in three variables *x*_1_, *x*_2_, *x*_3_



This function does not have any symmetry hyperplanes, but its interpolation  has degree 2.

### Theoretical results about steady-states

The following theorem investigates the steady-state behavior of discrete and continuous models.

**Theorem. ***Assume we are given a Boolean model and perfect continuous homologues **of the Boolean update functions. Then for any state vector **the following are equivalent:*

*(i) **is a steady-state of the Boolean model.*

*(ii) **is a steady-state of the model (1).*

(iii)  is a steady-state of the ODE model (2).

*Proof. *The steady-state conditions are



for the Boolean model,



for model (1) and



for model (2). Considering that the  are perfect homologues of the *B*_*i*_, these conditions are clearly equivalent.   □

*Remark. *Note that the above theorem generally applies to transformations using perfect continuous homologues of Boolean functions. In the special case of piecewise linear ODEs, i.e.  from equation (6), Glass et al. [11] could show that the above theorem also holds when *(iii) *is replaced by ' is a stable steady-state of the ODE model (2)'.

We now extend the above theorem to HillCube models. The problem is that we can no longer assume a perfect agreement between  and *B*_*i*_. The main idea is to use the implicit function theorem to prove the existence of a steady-state of the continuous model in a neighborhood of a Boolean steady-state.

**Theorem. ***Assume we are given a Boolean model and the corresponding HillCube model. Let x*^∞ ^∈ {0, 1}^*N *^*be a steady-state of the Boolean model. We fix the thresholds k*_*ij *_*at values in *(0, 1) *and the life-times τ*_*i *_*at values in *(0, ∞). *Then there exists a neighborhood U of x*^∞ ^*such that for all sufficiently large Hill exponents n *= (*n*_*ij*_) *the HillCube model has a stable steady-state **in U . It holds*



*Proof. *For a single Hill exponent *n *we write *n *= *m*^-2^, *m *≠ 0, fix some 0 <*k *< 1 and consider the Hill function



We extend this function at *m *= 0 by setting



Then there are open neighborhoods *U*_1_, *U*_2 _of 0 and 1, respectively, such that *f*(, *m*) is continuous on *U*_1/2 _× ℝ and it can be easily shown that *f *is even continuously differentiable on *U*_1/2 _× ℝ. Now consider the HillCube model. It depends only on the Hill exponents *n *and we define *m *= (*m*_*ij*_) by . For concentrations  let Φ(*m*, ) denote the right hand side of the HillCube ODE system. As explained above, we continuously extend the Hill functions and hence also Φ at *m *= 0. Then there exists an open neighborhood *U *⊂ ℝ^*N *^of *x*^∞ ^such that Φ is continuously differentiable on ℝ^|*m*| ^× *U *(as the composition of continuously differentiable functions).

For *m *= 0 the Hill functions become Boolean step functions and hence the HillCubes perfectly agree with the Boolean update rules. Therefore, we have Φ (0, *x*^∞^) = 0. Now, let us compute the Jacobian DΦ of Φ in (0, *x*^∞^).



where *δ*_*ij *_is the Kronecker delta. Note that for *m *= 0 the Hill functions in the HillCubes are step functions, i.e. constant in a neighborhood of *x*^∞^. Hence the derivative of the HillCubes vanishes. This shows that DΦ is diagonal negative definite and hence, in particular, invertible. Therefore, the implicit function theorem guarantees that there are open neighborhoods *U*^'^of *x*^∞ ^and *V' *⊂ ℝ^|*m*| ^of 0 such that for each *m *∈ *V'*, i.e. for large exponents *n*, there is a  ∈ *U' *such that Φ(*m*, ) = 0, i.e.  is a steady-state of the model. Since the mapping *m *↦  is continuous we have  → *x*^∞ ^as *m *→ 0 with respect to the euclidean norm, i.e. all *m*_*ij *_→ 0 or equivalently *n*_*ij *_→ ∞. Moreover, it follows that on subsets *V *⊆ *V' *and *U *⊆ *U' *the Jacobian of Φ is still negative definite and, consequently, the  are stable steady-states.   □

### Manual parameter determination for the continuous T-cell model

The question is how to encode the information about slow and fast interactions in the numeric values of the parameters. We illustrate our approach using the subnetwork shown in Figure 2C, where we focus on ZAP-70, LAT phosp and cCbl. The activation of LAT phosp stands for the interactions of scenario 2, whereas the activation of cCbl represents scenario 3. The ODE system for this network is given by



For the activation of ZAP-70 *k *= 0.3 and *τ*_*ZAP*-70 _= 10 are used. LAT phosp and cCbl are activated at thresholds *k*_fast _= 0.1 and *k*_slow _= 0.8, respectively, and both their life-times are set to *τ *= 1. Initial condition for all species is 0 and the inputs for ZAP-70 are fixed at 1. The result of the numeric simulation is shown in Figure 2D. From the beginning on ZAP-70 is activated but rather slowly due to the increased life-time *τ*_*ZAP*-70_. The activations of LAT-phosp and cCbl occur at around the time points when the concentration of ZAP-70 crosses the thresholds *k*_fast _and *k*_slow_, respectively. The high threshold *k*_slow _also leads to a lower total activation level of cCbl. One can clearly see the time lag between the activations of LAT phosp and cCbl, i.e. between the interactions in scenario 2 and the interactions not occurring until scenario 3.

### Model transformation and simulation

For the transformation of the Boolean T-cell model we choose HillCube ODEs. HillCubes are better suited to describe signaling cascades than BooleCubes. Normalization is not necessary as we will choose thresholds *k *≪ 1 and consequently the Hill functions will already satisfy *f*(1) ≈ 1. For the transformation and simulation we developed a MATLAB toolbox called *Odefy *[Krumsiek et al.: Odefy — From discrete to continuous models. In preparation (2009)], which is publicly available at  and allows the experimentalist to easily transform Boolean models into ODE models. Since *Odefy *can be integrated into *CellNetAnalyzer *[24], we were able to export the Boolean T-cell model from there. Numeric integration of the ODE system was carried out using MATLAB ode15s, a variable-order multistep solver based on the numerical differentiation formulas.

### Experimental data

Kemp et al. [12] created a data set describing the dynamics of the activation of the key signaling elements ERK, JNK, IKK and NFAT upon activation of the TCR. The data was generated by stimulation of a T-cell line (1B6 T cell hybridoma) with three peptides with different affinities for the T-cell receptor, Q144, Y144 and L144. In the case of L144 experiments were conducted for three different peptide concentrations 0.04 *μ*g/ml (low), 0.4 *μ*g/ml (medium) and 4 *μ*g/ml (high). In the case of Q144 and Y144 only the high concentration of 4 *μ*g/ml was used. Concentrations of ERK, JNK, IKK and NFAT were measured at 0, 10, 30, 60, 120, 240 and 2400 minutes. Here we neglected the last time point, as on this slow time scale many interactions play a role, that are not included in the model, such as gene expression. For the parameter fitting the data were linearly rescaled to the unit interval.

### Parameter fitting

The T-cell model consists of 40 species, 55 pairwise interactions and three external inputs. Hence, we have 40 life-time parameters and 58 pairs of Hill parameters, amounting to a total of 156 parameters. Due to this large number of parameters compared to the number of experimental data points, the fitting problem is obviously ill-posed as for many different parameter sets the model reproduces the data equally well. For this reason, we performed a two-step fitting process. First, we determined a parameter set for which the model fits the experimental data reasonably well. Second, we added a regularization to account for the indeterminacies. Both steps are optimization problems. The two cost functions are given below. They take a parameter set consisting of all Hill parameters (*n, k*) and all life-times *τ *as input a yield a scalar loss value, that needs to be minimized. In both steps we used a *simulated annealing *algorithm [25] for minimization. As the threshold parameters *k *have to be precisely adjusted at small values, these were fitted on a log-scale, as is also done in [26]. Parameters downstream of the measured species were, of course, not changed but fixed at their manually determined value. We used the SBPD package of the *Systems Biology Toolbox *[27] to create a compiled MATLAB simulation function of our ODE model for faster performance.

#### Least squares fitting

In the first step we determined a parameter set for which the model reproduces the data reasonably well. To this end, we employed a least squares fitting, i.e. we minimized the sum of the squared offsets of the data points from the model prediction. In a first attempt, we used only the offsets at the six time points *t *= 0, 10, 30, 60, 120, 240. This led to the model showing fast oscillations, which almost perfectly fitted the data, and, clearly, were an unrealistic overfitting. To avoid this, we linearly interpolated the experimental data and minimized the cost function



where  denotes the time courses predicted by the model and  the interpolated data points. The simulated annealing algorithm was started from the manually determined parameter set and finally converged at a cost function value of 18.98.

#### Regularization

In the regularization step we minimized the sum of the coefficients of variation *σ*_*n*_*/μ*_*n*_, *σ*_*k*_*/μ*_*k *_and *σ*_*τ*_*/μ*_*τ *_within the three parameter groups of *n*'s, *k*'s and *τ*'s, respectively, under the constraint that the model's fit to the data did not deteriorate. The corresponding cost function is



where the last term is a penalty term ensuring a constant quality of the model's fit. The simulated annealing was started from the result of the first step. Idea of this regularization is to account for parameter indeterminacies by reducing their variation and to enhance the significance of 'outliers' like the affinity *k*_*L*144 _of the TCR for the ligand L144, cf. Figure 4E.

Additional data file 6 shows model simulations for 5 different results of step 1. While not perfectly agreeing, their overall dynamic behavior is the same. We can reasonably assume that neither the parameter indeterminacies nor the regularization significantly influence the dynamics of the experimentally observed species.

## Authors' contributions

DMW conceived and designed the methodology, performed the computational experiments and drafted the manuscript. JK helped to perform the computational experiments and developed the computational platform. JSR, DAL and SK interpreted the numerical and experimental data and contributed to the manuscript. FJT participated in the design of the methodology and of the computational platform and helped to draft the manuscript. All authors read and approved the final manuscript.

## Supplementary Material

Additional file 1 **Example for the hypergraph representation of a Boolean model**. Supplementary text (.pdf) giving an example for the hypergraph representation of Boolean models. Click here for file

Additional file 2 **Hill functions**. Figure (.pdf) showing Hill functions *f*(*x*) = *x*^*n*^*/*(*x*^*n *^+ *k*^*n*^) with Hill coefficients *n *= 2, 4, 8, 16, 32 and threshold *k *= 0.5. Click here for file

Additional file 3 **HillCubes**. Figure (.png) showing HillCubes of all 16 two-variable Boolean gates. Hill parameters are *n *= 3 and *k *= 0.5 for both inputs. Click here for file

Additional file 4 **Regulatory domains**. Figure (.pdf) showing the regulatory domains in a two-variable example. Click here for file

Additional file 5 **Steady-states in discrete and continuous models**. Supplementary text (.pdf) discussing a toy example where the steady-states of a discrete model are not preserved in a continuous version of the model. Click here for file

Additional file 6 **Comparison of different best fit parameter sets with respect to model dynamics**. Figure (.pdf) showing simulations of the continuous T-cell model for 5 different best fit parameter sets (without regularization, cf. section on parameter fitting). While not perfectly agreeing, the overall dynamic behavior is the same in all simulations. Click here for file
