# Supplementary material for: Transforming Boolean models to continuous models: methodology and application to T-cell receptor signaling
Source: BMC Syst Biol. 2009 Sep 28;3:98. doi: 10.1186/1752-0509-3-98 (PMC2764636; doi:10.1186/1752-0509-3-98)
Supplement: Additional file 1 — Example for the hypergraph representation of a Boolean model. Supplementary text (.pdf) giving an example for the hypergraph representation of Boolean models. [file 1752-0509-3-98-S1.PDF]

Example for the hypergraph representation of a Boolean model

We consider the regulatory network between the three species X_1, X_2, X_3 given by the Boolean functions

$$\begin{aligned} B_1(x_2, x_3) &= x_2 \vee \neg x_3 \\ B_2(x_1, x_3) &= x_1 \wedge x_3 \\ B_3(x_1, x_2) &= x_1 \text{ XOR } x_2 \ . \end{aligned} \tag{1}$$

The corresponding truth tables are

	$x_2 = 0$	$x_2 = 1$
$x_3 = 0$	$B_1 = 1$	$B_1 = 1$
$x_3 = 1$	$B_1 = 0$	$B_1 = 1$

	$x_1 = 0$	$x_1 = 1$
$x_3 = 0$	$B_2 = 0$	$B_2 = 0$
$x_3 = 1$	$B_2 = 0$	$B_2 = 1$

	$x_1 = 0$	$x_1 = 1$
$x_2 = 0$	$B_3 = 0$	$B_3 = 1$
$x_2 = 1$	$B_3 = 1$	$B_3 = 0$

.

B_1 and B_2 are already in a sum-of-product form and B_3 can also be represented this way:

$$B_3(x_1, x_2) = (x_1 \wedge \neg x_2) \vee (\neg x_1 \wedge x_2) \ .$$

Figure 1 shows the hypergraph representing our network. Node X_1 has two incoming edges, a positive (black) one from x_2 and a negative (red) one from x_3. At node X_2 there is one incoming hyperedge representing the product $x_1 \wedge x_3$. Finally, we have two incoming hyperedges at node X_3 corresponding to the products $x_1 \wedge \neg x_2$ and $\neg x_1 \wedge x_2$.

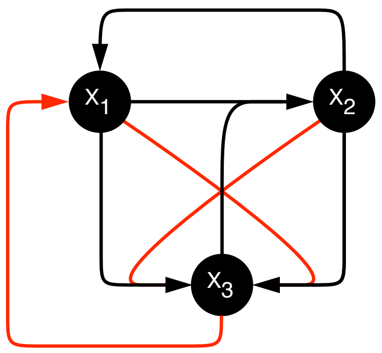

Figure 1: Hypergraph representation of model (1).
