# Supplementary figures and images for: Transforming Boolean models to continuous models: methodology and application to T-cell receptor signaling
Source: BMC Syst Biol. 2009 Sep 28;3:98. doi: 10.1186/1752-0509-3-98 (PMC2764636; doi:10.1186/1752-0509-3-98)

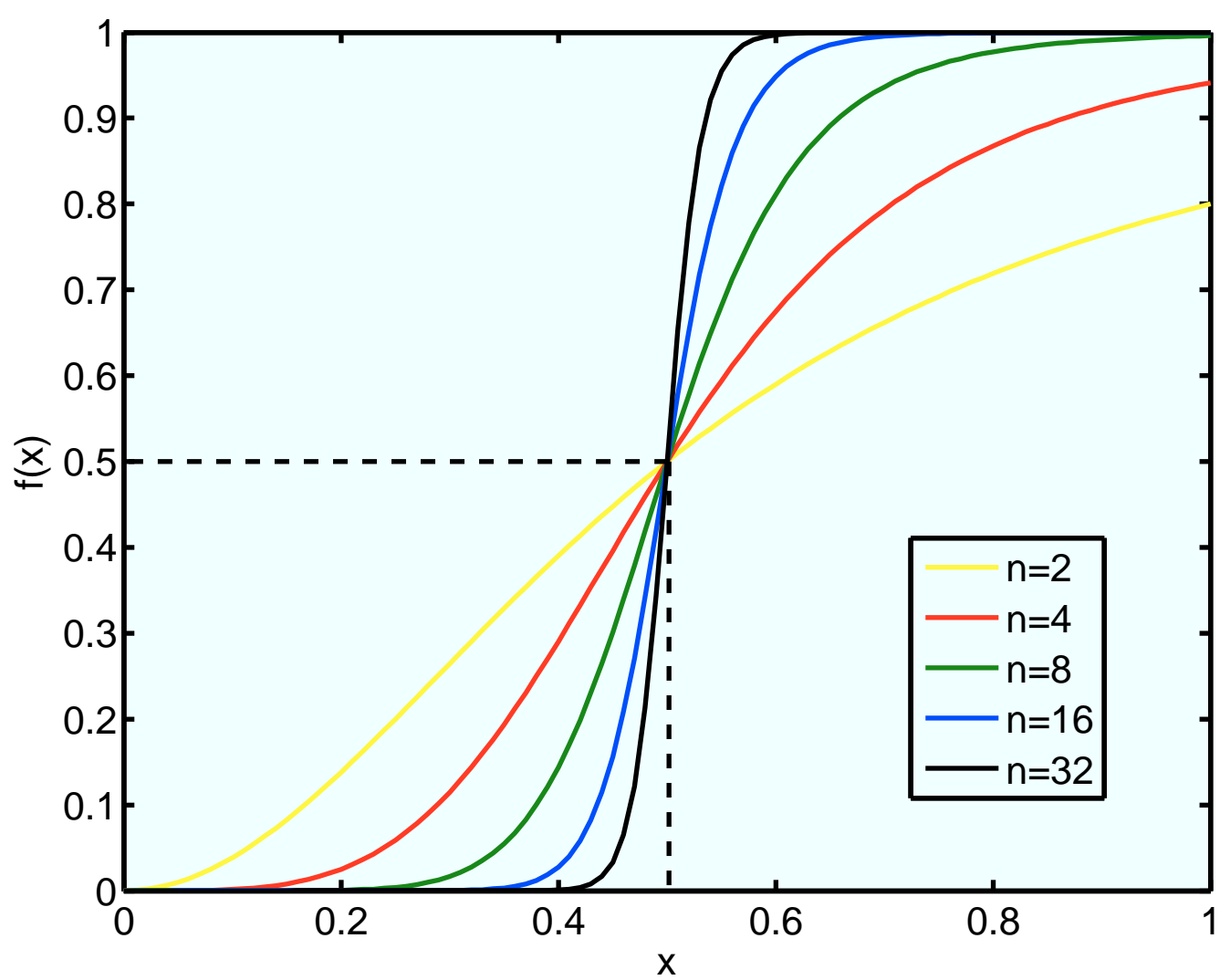

Supplement: Additional file 2 — Hill functions. Figure (.pdf) showing Hill functions f(x) = xn/(xn + kn) with Hill coefficients n = 2, 4, 8, 16, 32 and threshold k = 0.5. [file 1752-0509-3-98-S2.PDF]

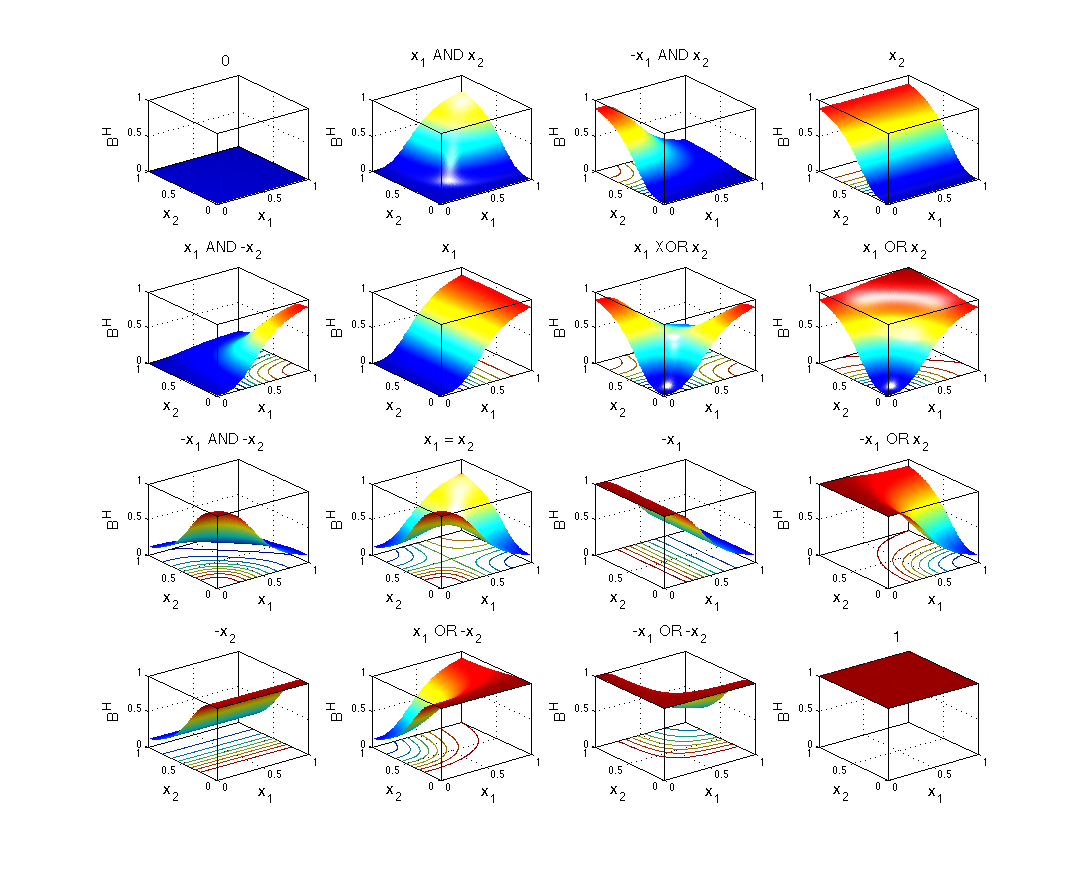

Supplement: Additional file 3 — HillCubes. Figure (.png) showing HillCubes of all 16 two-variable Boolean gates. Hill parameters are n = 3 and k = 0.5 for both inputs. [file 1752-0509-3-98-S3.PNG]

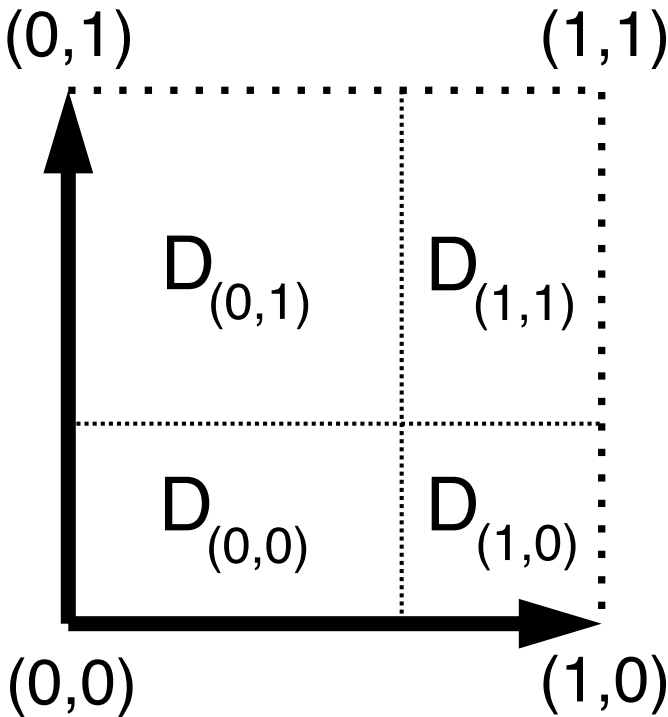

Supplement: Additional file 4 — Regulatory domains. Figure (.pdf) showing the regulatory domains in a two-variable example. [file 1752-0509-3-98-S4.PDF]

high

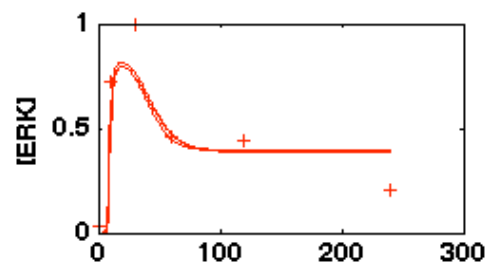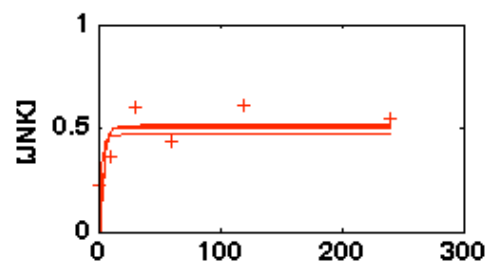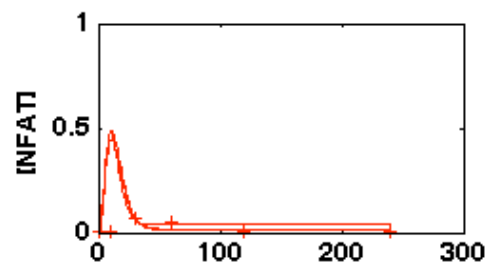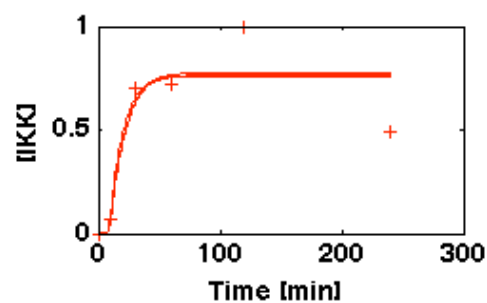

medium

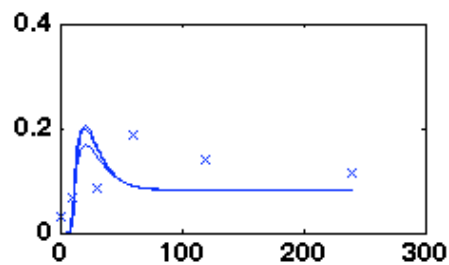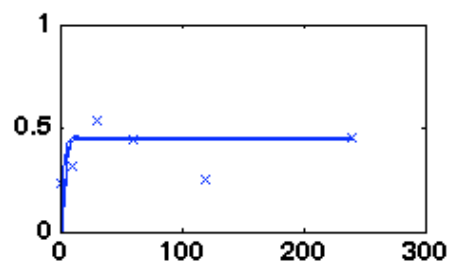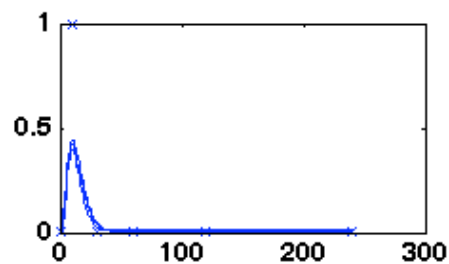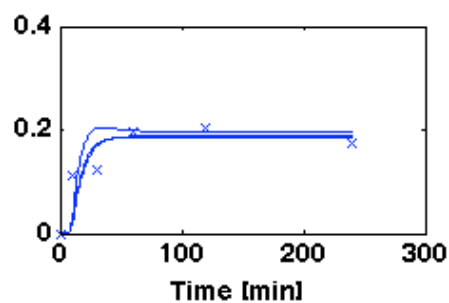

low

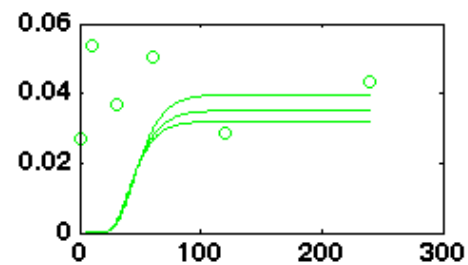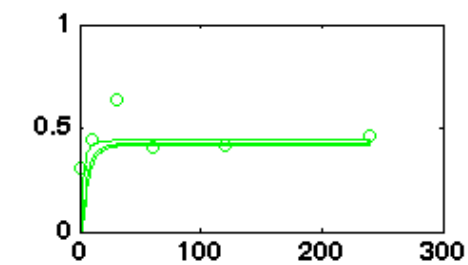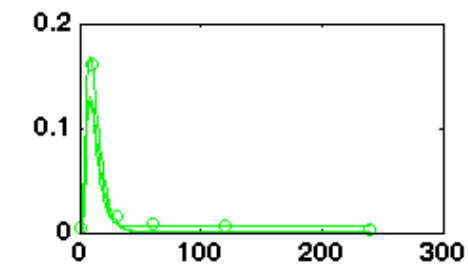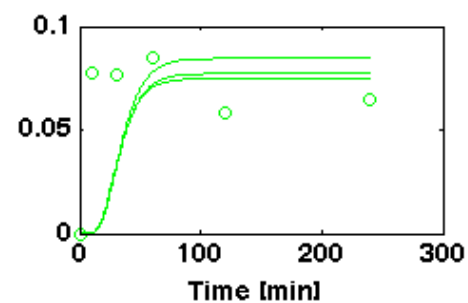

Supplement: Additional file 6 — Comparison of different best fit parameter sets with respect to model dynamics. Figure (.pdf) showing simulations of the continuous T-cell model for 5 different best fit parameter sets (without regularization, cf. section on parameter fitting). While not perfectly agreeing, the overall dynamic behavior is the same in all simulations. [file 1752-0509-3-98-S6.PDF]
