# Supplementary material for: Transforming Boolean models to continuous models: methodology and application to T-cell receptor signaling
Source: BMC Syst Biol. 2009 Sep 28;3:98. doi: 10.1186/1752-0509-3-98 (PMC2764636; doi:10.1186/1752-0509-3-98)
Supplement: Additional file 5 — Steady-states in discrete and continuous models. Supplementary text (.pdf) discussing a toy example where the steady-states of a discrete model are not preserved in a continuous version of the model. [file 1752-0509-3-98-S5.PDF]

Steady states in discrete and continuous models

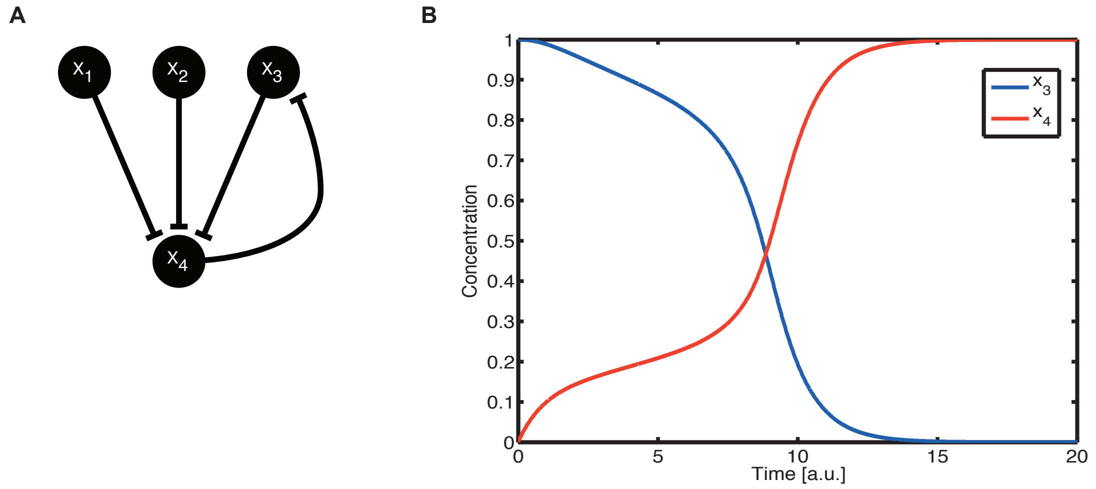

We consider the regulatory network between the species X_1, X_2, X_3, X_4 from Figure A, where multiple inputs at a node are linked by an AND gate. Note that this is not the hypergraph representation of the sum-of-product form. The corresponding update functions for species X_3 and X_4

$$B_3(x_4) = \neg x_4 \quad \text{and}$$
$$B_4(x_1, x_2, x_3) = \neg\,(x_1 \vee x_2 \vee x_3)$$

are chosen to belong to the limited set of Boolean functions allowed in [1]. The state vector $x_1 = 0, x_2 = 0, x_3 = 1, x_4 = 0$ is a steady state of the Boolean model. The presence of one of its inhibitors (X_3) is enough to repress X_4. X_3 in turn is active since its only inhibitor (X_4) is off. Note that this is exactly the kind of logic used in [1]. We can now build a continuous model using the transformation method introduced in [1]. The inputs to nodes X_3 and X_4 are

$$\omega_3(\overline{x}_4) = 1 - 2\frac{\overline{x}_4}{1+\overline{x}_4} \quad \text{and}$$
$$\omega_4(\overline{x}_1, \overline{x}_2, \overline{x}_3) = 1 - \frac{4}{3}\frac{\overline{x}_1 + \overline{x}_2 + \overline{x}_3}{1 + \overline{x}_1 + \overline{x}_2 + \overline{x}_3}\ .$$

We have $\omega_3(0) = 1$ and $\omega_3(1) = 0$, as expected. However, the input to node X_4 is only minimal (0) iff $\overline{x}_1 = \overline{x}_2 = \overline{x}_3 = 1$, which does not agree with the Boolean logic. Here, $B_4(x_1, x_2, x_3) = 0$ iff at least one inhibitor is active.

We now run a numeric simulation of the continuous model starting at the Boolean steady state $\overline{x}_1 = 0, \overline{x}_2 = 0, \overline{x}_3 = 1, \overline{x}_4 = 0$, cf. Figure B. While node X_4 should be completely repressed in this steady state, it still receives an input of $\omega_4 = 1/3$ in the ODE model, which is too high to permanently inhibit its activation. Consequently, we observe a switching of X_3 and X_4 and the model ultimately comes to a rest in the steady state $\overline{x}_1 = 0, \overline{x}_2 = 0, \overline{x}_3 = 0, \overline{x}_4 = 1$.

References

[1] Luis Mendoza and Ioannis Xenarios. A method for the generation of standardized qualitative dynamical systems of regulatory networks. *Theoretical Biology and Medical Modelling*, 3(13), 2006.
